# Inhibitors of enhancer of zeste homolog 2 (EZH2) activate tumor-suppressor microRNAs in human cancer cells

**DOI:** 10.1038/oncsis.2014.17

**Published:** 2014-05-26

**Authors:** S Hibino, Y Saito, T Muramatsu, A Otani, Y Kasai, M Kimura, H Saito

**Affiliations:** 1 Division of Pharmacotherapeutics, Keio University Faculty of Pharmacy, Minato-ku, Tokyo, Japan

**Keywords:** EZH2, microRNA, SAHA, DZNep, cancer

## Abstract

Enhancer of zeste homolog 2 (EZH2) enhances tumorigenesis and is commonly overexpressed in several types of cancer. To investigate the anticancer effects of EZH2 inhibitors, microRNA (miRNA) expression profiles were examined in gastric and liver cancer cells treated with suberoylanilide hydroxamic acid (SAHA) and 3-deazaneplanocin A (DZNep). We confirmed that SAHA and DZNep suppressed EZH2 expression in AGS and HepG2 cells and inhibited their proliferation. The results of microarray analyses demonstrated that *miR-1246* was commonly upregulated in cancer cells by treatment with SAHA and DZNep. *MiR-302a* and *miR-4448* were markedly upregulated by treatment with SAHA and DZNep, respectively. DYRK1A, CDK2, BMI-1 and Girdin, which are targets of *miR-1246*, *miR-302a* and *miR-4448*, were suppressed by treatment with SAHA and DZNep, leading to apoptosis, cell cycle arrest and reduced migration of AGS and HepG2 cells. ChIP assay revealed that SAHA and DZNep inhibited the binding of EZH2 to the promoter regions of *miR-1246*, *miR-302a* and *miR-4448*. These findings suggest that EZH2 inhibitors such as SAHA and DZNep exert multiple anticancer effects through activation of tumor-suppressor miRNAs.

## Introduction

Epigenetic silencing of tumor-suppressor genes in human cancer is mediated by aberrant DNA methylation and histone modification. The polycomb repressive complex 2 mediates epigenetic gene silencing by trimethylating histone H3 lysine 27 and is known to aberrantly silence tumor-suppressor genes in cancer. Enhancer of zeste homolog 2 (EZH2), which is the catalytic subunit of polycomb repressive complex 2, enhances tumorigenesis and is commonly overexpressed in several types of cancer.^1^ Moreover, EZH2 has been reported to have an essential role in self-renewal of cancer stem cells,^2^ indicating that EZH2-targeting drugs can act as potent anticancer agents.

Epigenetic therapy with DNA methylation inhibitors and histone-modifying drugs has emerged as an effective approach for chemotherapy as well as chemoprevention of cancer. The histone deacetylase (HDAC) inhibitor suberoylanilide hydroxamic acid (SAHA) has been approved for patients with cutaneous T-cell lymphoma.^3^ Recently, it was discovered that 3-deazaneplanocin A (DZNep) inhibits EZH2, which has H3K27 trimethylation activity.^4^ Although SAHA is widely accepted as an HDAC inhibitor, it has been reported to suppress EZH2 expression in cancer cells and to exert an anticancer effect, indicating that SAHA also functions as an EZH2 inhibitor.^5, 6^

MicroRNAs (miRNAs) are small noncoding RNAs that function as endogenous silencers of various target genes. Specific miRNAs such as *miR-34a* are downregulated in various cancers and act as tumor suppressors. On the other hand, miRNAs such as *miR-155* and the *miR-17-92* cluster are reportedly overexpressed in various cancers, and act as oncogenes.^7, 8, 9, 10, 11^ Aberrant expression of miRNAs has a critical role in human carcinogenesis. We have discovered that some miRNAs including *miR-127* are regulated by epigenetic alterations such as DNA methylation and histone modification.^12^ DNA methylation inhibitors and HDAC inhibitors can activate epigenetically silenced tumor-suppressor miRNAs accompanied by downregulation of target oncogenes in human cancer cells.^12, 13^ However, the miRNA expression profiles altered by EZH2 inhibitors are still unknown. In the present study, to investigate the molecular mechanisms underlying the anticancer effects of EZH2 inhibitors, miRNA expression profiles in gastric and liver cancer cells were analyzed after treatment with SAHA and DZNep.

## Results

### SAHA and DZNep inhibit EZH2 expression in, and proliferation of, AGS and HepG2 cells

**Figure 1:**
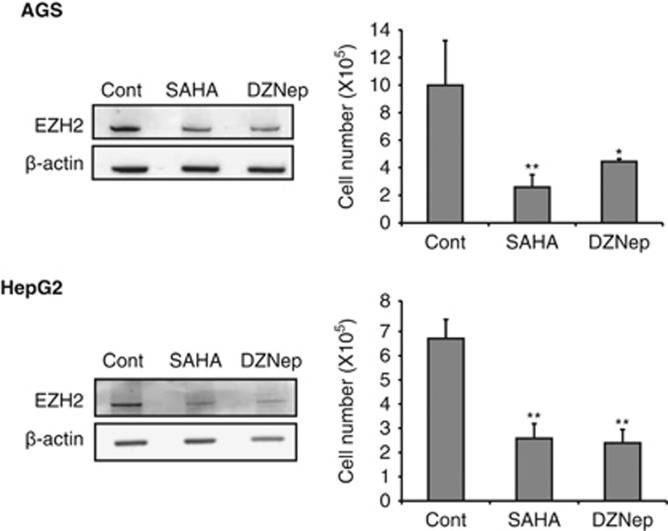
EZH2 expression and proliferation of cancer cells treated with SAHA and DZNep. Western blotting of EZH2 and cell counting assay were performed in AGS and HepG2 cells treated with SAHA and DZNep. *P<0.01, **P<0.001.

We first investigated the levels of EZH2 expression and the antiproliferative activity of SAHA and DZNep in AGS and HepG2 cells. As shown in Figure 1, EZH2 expression in both AGS and HepG2 cells was suppressed by treatment with 1 μM SAHA and 5 μM DZNep for 72 h. The numbers of AGS and HepG2 cells were significantly reduced 72 h after treatment with SAHA and DZNep. These findings suggest that both AGS and HepG2 cells are sensitive to SAHA and DZNep, and that these histone-modifying drugs inhibit EZN2 expression and the proliferative activity of cancer cells derived from the stomach and the liver.

**Figure 2:**
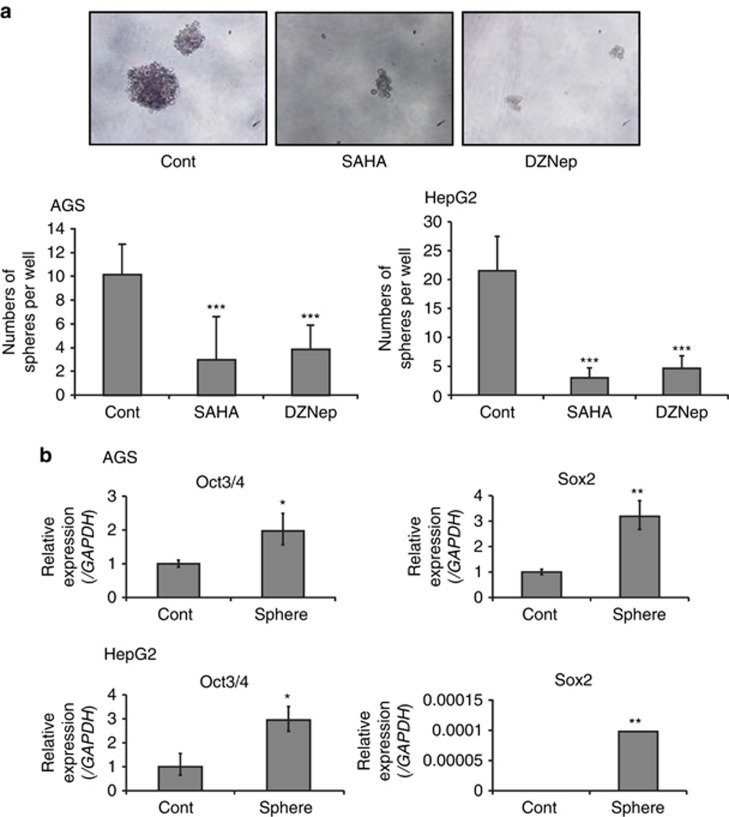
Alteration of stemness in cancer cells after treatment with EZH2 inhibitors. (**a**) Sphere-forming assay of AGS and HepG2 cells treated with SAHA and DZNep. Under anchorage-independent stem cell-specific culture conditions, AGS and HepG2 cells form spheroids. Spheroids derived from AGS cells are shown. The number of spheres derived from AGS and HepG2 cells treated with SAHA and DZNep was compared with control cells. ***P<0.001. (**b**) Quantitative RT-PCR of *Oct3/4* and *Sox2* in spheroids of AGS and HepG2 cells. Relative expression of *Oct3/4* and *Sox2* in spheres derived from AGS and HepG2 cells was compared with that of control cells. For control of *Oct3/4* and *Sox2* expression after spheroid formation, we used AGS or HepG2 cells cultured in regular media supplemented with 10% FBS. *P<0.05, **P<0.01.

We also examined the alteration of stemness in AGS and HepG2 cells using a sphere-forming assay. Under anchorage-independent stem cell-specific culture conditions, AGS and HepG2 cells formed spheroids, as shown in Figure 2a. Treatment with SAHA and DZNep significantly reduced the number of these spheroids. The levels of expression of *Oct3/4* and *Sox2*, which are essential for induction of pluripotent stem (iPS) cells, were significantly increased in AGS and HepG2 cells after spheroid formation. These results suggest that SAHA and DZNep suppress the stemness of AGS and HepG2 cells (Figure 2b).

### *miR-1246* is a common target of EZH2 inhibitors in cancer cells

**Figure 3:**
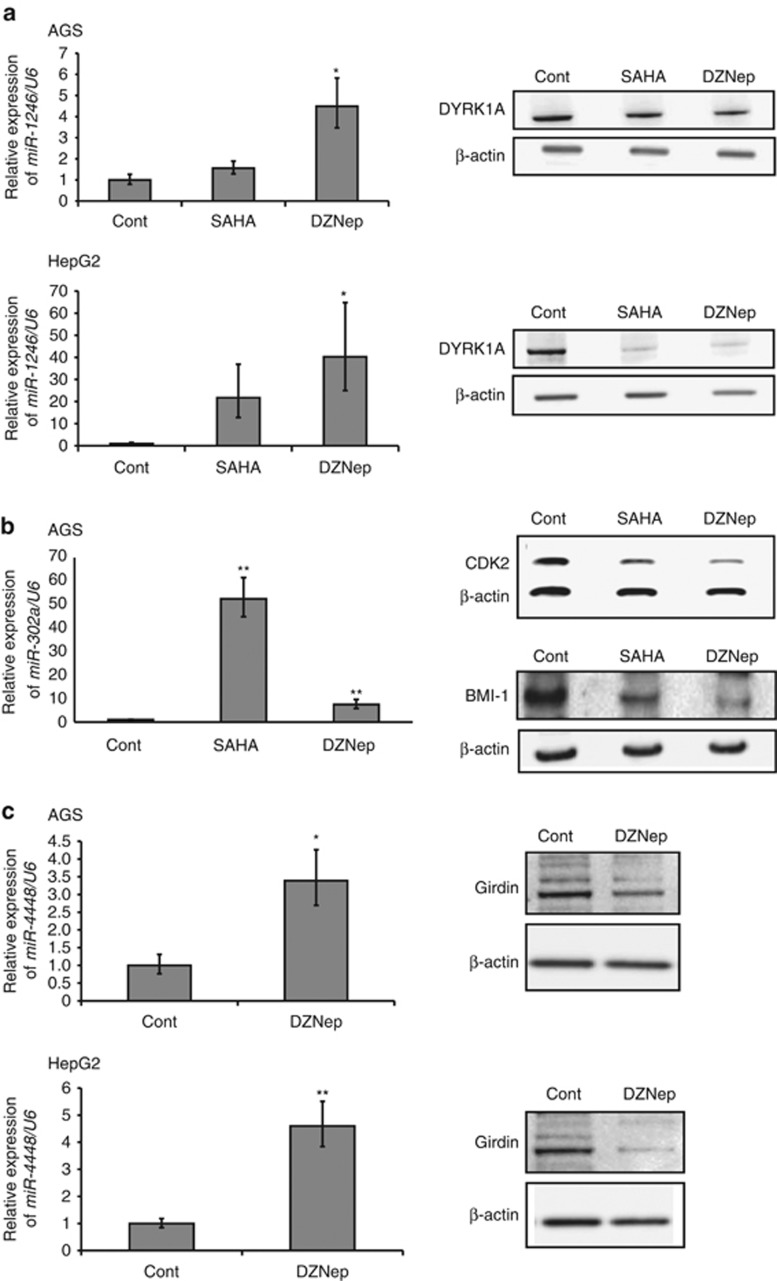
Expression levels of *miR-1246*, *miR-302a* and *miR-4448* and their target genes in AGS and HepG2 cells treated with EZH2 inhibitors. (**a**) Quantitative RT-PCR of *miR-1246* and western blotting of DYRK1A in AGS and HepG2 cells treated with SAHA and DZNep. *P<0.05. (**b**) Quantitative RT-PCR of *miR-302a* and western blotting of CDK2 and BMI-1 in AGS cells treated with SAHA and DZNep. **P<0.01. (**c**) Quantitative RT-PCR of *miR-4448* and western blotting of Girdin in AGS and HepG2 cells treated with DZNep. *P<0.05, **P<0.01.

To investigate the miRNA expression profiles altered by the treatment of AGS and HepG2 cells with SAHA and DZNep, we conducted microarray analyses. miRNAs that were significantly upregulated after treatment of AGS and HepG2 cells with SAHA and DZNep are summarized in Table 1. Interestingly, *miR-1246* was upregulated in both cell lines after SAHA and DZNep treatment (Table 1). Increased expression of *miR-1246* by treatment with SAHA and DZNep was confirmed by quantitative RT–PCR (Figure 3a). This suggests that *miR-1246* is a common target of SAHA and DZNep in cancer cells and can be activated by these histone-modifying drugs.

### *miR-302a* and *miR-4448* are upregulated by SAHA and DZNep

We also found that *miR-302a* and *miR-4448* were most upregulated by treatment of AGS cells with SAHA, and by treatment of both AGS and HepG2 cells with DZNep, respectively (Table 1). Upregulation of *miR-302a* and *miR-4448* by treatment with SAHA and DZNep was confirmed by quantitative RT–PCR (Figures 2b and c). Recent studies have shown that *miR-302* is the major miRNA found in human embryonic stem cells and iPS cells, and that induction of *miR-302* expression reprograms somatic cells into a pluripotent stem cell-like state.^14, 15^ *MiR-302* has been reported to inhibit the tumorigenicity of human pluripotent stem cells and the proliferation of cervical carcinoma cells.^16, 17^ Although *miR-4448* was identified only recently and its function is still unclear, we focused our study on *miR-4448*, as it was robustly induced by DZNep treatment.

### *miR-1246*, *miR-302a* and *miR-4448* suppress their target genes upon treatment with SAHA and DZNep in cancer cells

A recent study has shown that dual-specificity tyrosine phosphorylation-regulated kinase 1A (DYRK1A), a Down syndrome-associated protein kinase, is a target of *miR-1246*.^18^ We examined the levels of expression of *miR-1246* and its target DYRK1A by quantitative RT–PCR and western blotting, respectively. As shown in Figure 3a, the expression level of *miR-1246* was increased, and accompanied by downregulation of DYRK1A, after treatment of AGS and HepG2 cells with SAHA and DZNep. Cyclin-dependent kinase 2 (CDK2) and BMI-1 polycomb ring finger oncogene (BMI-1), both of which are known to be cell cycle regulators, have been identified as targets of *miR-302.*^16^ Figure 3b shows the expression levels of *miR-302a* and its targets CDK2 and BMI-1. *MiR-302a* was significantly upregulated in comparison with control cells, and CDK2 and BMI-1 were downregulated, after treatment of AGS cells with SAHA and DZNep.

To investigate whether *miR-4448* targets any cancer-related genes, we searched the miRNA-target databases Targetscan (http://www.targetscan.org/vert_61/) and miRDB (http://mirdb.org/miRDB/). These databases strongly implicate CCDC88A (coiled-coil domain containing 88A, also known as Girdin) as a candidate target gene of *miR-4448* with high scores. Girdin is a component of the phosphatidylinositol 3-kinase (PI3-K)/Akt pathway, which is a pivotal signaling pathway for cancer progression and has an important role in cancer cell migration by controlling actin organization.^19^ As shown in Figure 3c, expression of Girdin protein was reduced after DZNep treatment in both AGS and HepG2 cells, and this was accompanied by upregulation of *miR-4448* expression.

### SAHA and DZNep induce apoptosis and cell cycle (G1/S) arrest in AGS and HepG2 cells and inhibit their migration

**Figure 4:**
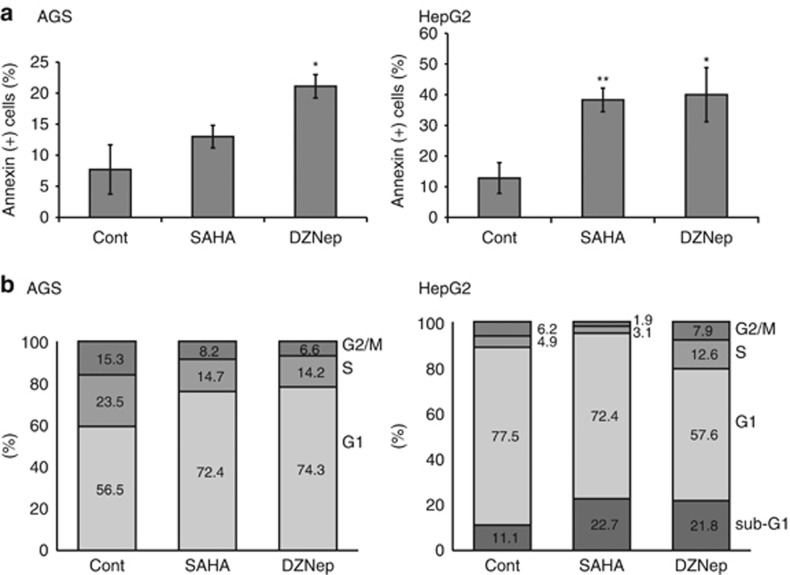
Apoptosis and cell cycle assays of AGS and HepG2 cells treated with EZH2 inhibitors. (**a**) Annexin V apoptosis assay of AGS and HepG2 cells treated with SAHA and DZNep. *P<0.01, **P<0.001. (**b**) Cell cycle assay of AGS and HepG2 cells treated with SAHA and DZNep.

To elucidate the molecular mechanism underlying the suppression of cancer cells by histone-modifying drugs, we conducted Annexin V-FITC apoptosis assay and cell cycle assay. As shown in Figure 4a, flow cytometry analyses revealed that Annexin V-positive cells were increased among both AGS and HepG2 cells after treatment with SAHA and DZNep, indicating that these histone-modifying drugs induce apoptosis of cancer cells. Figure 4b shows the results of the cell cycle assay in which AGS and HepG2 cells were treated with histone-modifying drugs. The proportion of AGS cells in G1-phase was increased after treatment with SAHA and DZNep, suggesting induction of G1/S arrest. On the other hand, the proportion of HepG2 cells in sub-G1-phase was increased after treatment with SAHA and DZNep, suggesting induction of apoptosis by these drugs.

**Figure 5:**
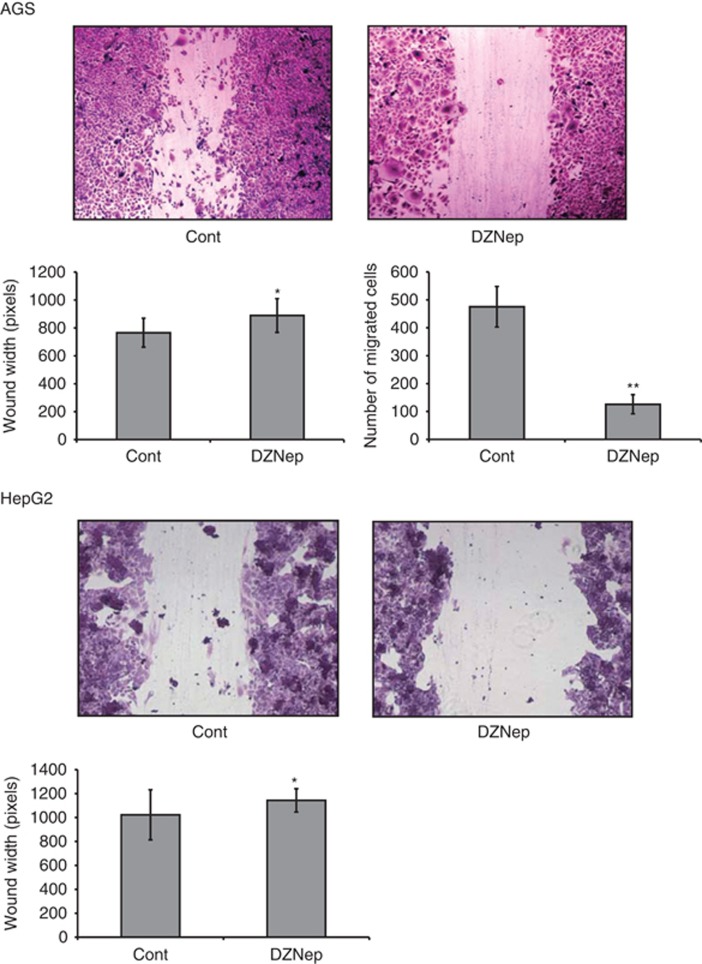
Wound-healing assay of AGS and HepG2 cell migration after treatment with DZNep. Wound healing of AGS and HepG2 cells is shown. Wound width of plated AGS and HepG2 cells after DZNep treatment was compared with that of control cells. The number of migrated AGS cells after treatment with DZNep was compared with that of control cells. *P<0.05, **P<0.01.

As Girdin, which is associated with cancer cell migration, was downregulated by DZNep treatment, we conducted the wound-healing assay, which is a standard assay for measuring cell migration. As shown in Figure 5, after DZNep treatment, the wound width of plated AGS and HepG2 cells was significantly increased, and the number of migrated AGS cells was significantly suppressed. These results suggest that DZNep suppresses the migration of AGS and HepG2 cells.

### Binding of EZH2 to the promoter regions of *miR-1246*, *miR-302a* and *miR-4448* is inhibited by SAHA and DZNep

**Figure 6:**
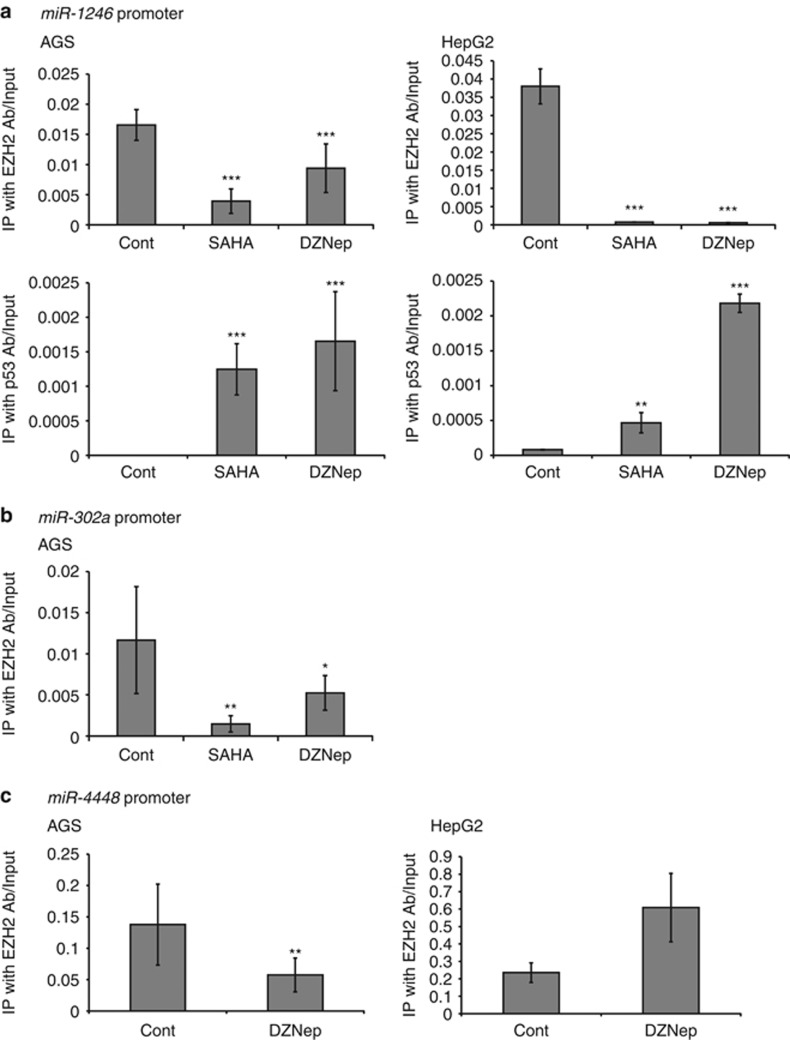
ChIP assays of binding of anti-EZH2 and -p53 antibodies to the promoter regions of *miR-1246, miR-302a and miR-4448.* (**a**) ChIP assay to determine binding of antibodies against EZH2 and p53 to the promoter region of *miR-1246* in AGS and HepG2 cells treated with SAHA and DZNep. **P<0.01, ***P<0.001. (**b**) ChIP assay to determine binding of anti-EZH2 antibody to the promoter region of *miR-302a* in AGS cells treated with SAHA and DZNep. *P<0.05, **P<0.01. (**c**) ChIP assay to determine binding of anti-EZH2 antibody to the putative promoter region of *miR-4448* in AGS and HepG2 cells treated with DZNep. **P<0.01.

Finally, we performed the ChIP assay with antibodies against EZH2 and p53 to clarify the mechanism responsible for regulation of these miRNAs by histone-modifying drugs. Chromatin around the *miR-1246* promoter region that was immunoprecipitated with EZH2 antibody was significantly reduced by treatment with SAHA and DZNep in both AGS and HepG2 cells (Figure 6a). A recent study has shown that *miR-1246* has a p53-responsive element in its promoter region, and that p53 induces *miR-1246* expression in response to DNA damage.^18^ The results of the ChIP assay demonstrate that binding of p53 to the *miR-1246* promoter region was significantly increased by treatment of cancer cells with SAHA and DZNep (Figure 6a).

Chromatin around the *miR-302a* promoter region that was immunoprecipitated with EZH2 antibody was significantly reduced by treatment with SAHA and DZNep in AGS cells (Figure 6b). As for *miR-4448*, we examined the potential promoter region located on chromosome 3: 183602504-183602724. Binding of EZH2 to the potential *miR-4448* promoter was significantly decreased after DZNep treatment in AGS cells. Unexpectedly, in HepG2 cells, binding of EZH2 was increased after DZNep treatment (Figure 6c). These findings indicate that binding of EZH2 to the promoter regions of *miR-1246*, *miR-302a* and *miR-4448* was inhibited in cancer cells by treatment with SAHA and DZNep.

## Discussion

Drugs that target chromatin-modifying enzymes such as DNA methylation inhibitors and HDAC inhibitors hold clinical promise for treatment of cancer. However, the molecular mechanisms underlying the anticancer effects of EZH2 inhibitors are not fully understood. DZNep inhibits EZH2, which exerts trimethylation activity on H3K27. SAHA is widely accepted as an HDAC inhibitor, but its function as an EZH2 inhibitor has also been reported.^5, 6^ Recent studies have shown that EZH2 interacts with HDACs via EED (embryonic ectoderm development: as one of the polycomb repressive complex 2 component proteins) and that EZH2-mediated gene silencing is dependent on HDAC activity.^20^ In addition, another HDAC inhibitor, trichostatin A, completely abrogates the effects of EZH2 overexpression in cancer cells, thus supporting the function of SAHA as an EZH2 inhibitor.^21^

Here we have shown for the first time that, in cancer cells, *miR-1246* is a common target of EZH2 inhibitors and that *miR-302a* and *miR-4448* are activated by SAHA and DZNep. Our results demonstrate that SAHA and DZNep suppress EZH2 expression and activate tumor-suppressor miRNAs in cancer cells. ChIP assay revealed that binding of EZH2 to the promoter regions of *miR-1246*, *miR-302a* and *miR-4448* was inhibited by SAHA and DZNep. As EZH2 is the catalytic subunit of polycomb repressive complex 2, which mediates epigenetic gene silencing by trimethylating histone H3 lysine 27, suppression of EZH2 by SAHA and DZNep may induce transcriptional activation of *miR-1246*, *miR-302a* and *miR-4448* in cancer cells. In HepG2 cells, binding of EZH2 to the putative *miR-4448* promoter region was not suppressed by DZNep, suggesting that *miR-4448* expression is not under the direct control of EZH2. Transcription factors regulated by EZH2 may control *miR-4448* expression in liver cancer cells. In addition, *miR-1246* has a p53-responsive element in its promoter region, and p53 induces *miR-1246* expression.^18^ Treatment with SAHA and DZNep increased p53 binding to the *miR-1246* promoter region, thus further increasing *miR-1246* expression.

DYRK1A, which was recently identified as one of the targets of *miR-1246*, was significantly suppressed by treatment with EZH2 inhibitors, resulting in apoptosis of AGS and HepG2 cells. DYRK1A has been shown to suppress caspase-9-mediated apoptosis in mammalian cells through phosphorylation of caspase-9 on threonine residue 125.^22, 23^ These findings suggest that SAHA and DZNep induce DYRK1A-mediated apoptosis in cancer cells through activation of *miR-1246*. Treatment of AGS cells with DZNep and SAHA suppressed CDK2 and BMI-1, which were recently identified as the targets of *miR-302*, and induced cell cycle (G1/S) arrest. CDK2 is a G1-phase checkpoint regulator, and its suppression may result in inhibition of G1–S cell cycle transition pathways. Deficiency of BMI-1, an oncogenic cancer stem cell marker, has been shown to inhibit G1–S cell cycle transition through enhancement of p16Ink4a and p14Arf tumor-suppressor activities.^24^ Thus, concurrent silencing of CDK2 and BMI-1 by *miR-302a* may synergistically bring about G1/S cell cycle arrest of AGS cells. We also found that treatment with DZNep suppressed the expression of Girdin and inhibited the migration of AGS and HepG2 cells, probably because of the activation of *miR-4448*. It has been reported previously that Girdin is highly expressed in a variety of human cancers and that shRNA knockdown of Girdin markedly inhibits the metastasis of breast cancer cells.^25^ These findings suggest that DZNep may impair progression of cancer and migration of cancer cells by suppression of Girdin through activation of *miR-4448*.

In the present study, we focused on three miRNAs—*miR-1246*, *miR-302a* and *miR-4448*—which were robustly upregulated by SAHA and DZNep treatment in AGS and HepG2 cells. However, other miRNAs listed in Table 1 are also associated with apoptosis and migration of cancer cells. For example, it has been reported that *miR-15, miR-16* and *miR-181a* induce apoptosis of cancer cells by targeting *BCL2*^26, 27, 28^ and that *miR-205* inhibits the migration of cancer cells.^29, 30^ In addition, recent studies have shown that *miR-1915* also targets *BCL2* and has an important role in the development of multidrug resistance in colorectal carcinoma cells^31^ and that *miR-146b-5p* reduces migration and invasion of glioma by targeting epidermal growth factor receptor and matrix metalloproteinase 16.^32, 33^ On the other hand, several potential oncogenic miRNAs such as *miR-21*, *miR-103* and *miR-221* are also upregulated after treatment with SAHA and DZNep.^34, 35, 36, 37^ In particular, *miR-221* promotes tumorigenesis of gastric and liver cancer cells by targeting tumor-suppressor genes such as *PTEN*.^35, 36^ Gebeshuber *et al.*^38^ have proposed that whether one miRNA acts as a tumor suppressor or oncogene is largely dependent on cell condition. Further studies are needed to examine how these potential oncogenic miRNAs are implicated in the anticancer effect of EZH2 inhibitors.

As EZH2 has been reported to be essential for self-renewal of cancer stem cells,^2^ SAHA and DZNep, which inhibit EZH2, could be promising therapeutic agents for suppression of cancer stem cells.^39, 40^ After culture of AGS and HepG2 cells under anchorage-independent stem cell-specific conditions, spheroids were formed, as shown in Figure 2a. The stem cell properties of the spheroid-forming cells were confirmed by upregulation of *Oct3/4* and *Sox2*, which are essential for iPS cells. SAHA and DZNep significantly inhibited spheroid formation, suggesting that EZH2 inhibitors suppress the stemness of AGS and HepG2 cells. Other studies have also shown that EZH2 inhibitors act against the cancer stem cell population in pancreatic cancer and glioblastoma.^39, 40, 41^ These findings indicate that EZH2 inhibitors may be promising agents for cancer stem cell-targeting therapy.

Accumulating evidence has revealed that the *miR-302-367* cluster is deeply associated with stemness in pluripotent cells, but we and other groups have shown that the *miR-302-367* cluster also acts as a potent tumor suppressor in cancer stem cells. Fareh *et al.*^42^ have reported that the *miR-302-367* cluster has an ability to suppress the cancer-stemness signature of glioma-initiating cells. In addition, recent studies have reported that the *miR-520* cluster, which is overexpressed in human ES cells, also acts as a tumor suppressor, and that introduction of *miR-520h* mimics into pancreatic cancer cells results in reduction of side population cells.^43, 44^ In general, miRNAs have multiple functions in response to different cell conditions through suppression of various target genes. Our results suggest that *miR-302a* acts as a tumor suppressor and suppresses the cancer-stemness signature in cancer cells by suppressing target genes such as *CDK2* and *BMI-1*, although it has an important role in maintaining stemness in pluripotent cells such as ES cells and iPS cells.

**Figure 7:**
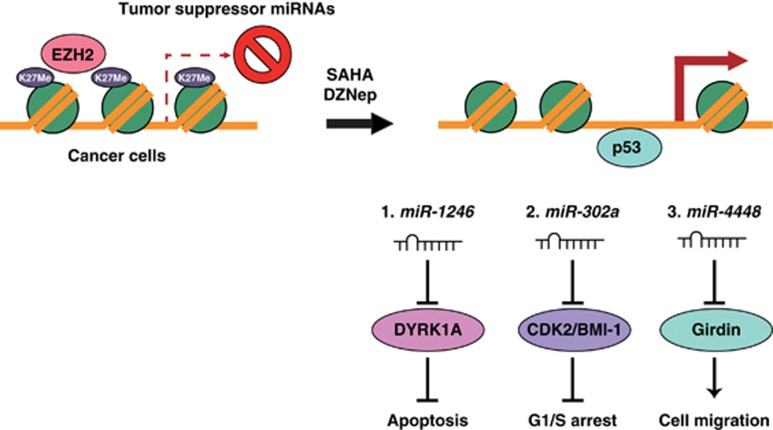
A model summarizing activation of tumor-suppressor miRNAs by SAHA and DZNep. In cancer cells, tumor-suppressor miRNAs are silenced by a repressive chromatin structure involving H3K27me3 mediated by chromatin-modifying factors including EZH2. Treatment with SAHA and/or DZNep suppresses EZH2 expression and reduces the level of H3K27 methylation, leading to creation of an active chromatin structure and allowing p53 to bind to the promoter region of *miR-1246*. Tumor-suppressor miRNAs such as *miR-1246*, *miR-302a* and *miR-4448* are activated and suppress their cancer-related target genes, resulting in induction of apoptosis and G1/S arrest in cancer cells and inhibition of their migration.

Figure 7 summarizes our present findings. In cancer cells, tumor-suppressor miRNAs are silenced by a repressive chromatin structure involving trimethylating histone H3 lysine 27 mediated by chromatin-modifying factors including EZH2. Treatment with SAHA and/or DZNep suppresses EZH2 expression and reduces the level of H3K27 methylation, creating a more active chromatin structure, and thus allowing p53 to bind to the promoter region of *miR-1246*. Tumor-suppressor miRNAs such as *miR-1246*, *miR-302a* and *miR-4448* are activated and suppress their cancer-related target genes, thus inducing apoptosis and G1/S arrest of cancer cells and inhibiting their migration. In conclusion, EZH2 inhibitors such as SAHA and DZNep exert multiple anticancer effects through activation of tumor-suppressor miRNAs in human cancer cells. Epigenetic therapy with EZH2 inhibitors holds clinical promise for the management of human malignancies.

## Materials and methods

### Cell culture and drug treatment

AGS, a human gastric cancer cell line, was obtained from the American Type Culture Collection (Rockville, MD, USA) and cultured in RPMI1640 medium supplemented with 10% fetal bovine serum (FBS). HepG2, a human liver cancer cell line, was obtained from RIKEN Cell Bank (Tsukuba, Japan) and cultured in Dulbecco's Minimal Essential Medium supplemented with 10% FBS. For drug treatment, cells were seeded the day before drug administration, and then treated with 5 μM DZNep (Cayman Chemical, Ann Arbor, MI, USA) or 1 μM SAHA (Sigma-Aldrich, St Louis, MO, USA) for 72 h.^45, 46^

### Cell counting

AGS and HepG2 cells were seeded at 1.0 × 10^5^ cells per well in a 6-well plate. AGS and HepG2 cells were cultured in media supplemented with 10% FBS and treated with 1 μM SAHA and 5 μM DZNep for 72 h. After treatment with DZNep and SAHA, the cells were counted using a Cellometer (Tomy Digital Biology, Tokyo, Japan). The data are indicated as mean±s.d. from three independent experiments.

### RNA extraction and microarray analysis

Total RNAs were extracted from cancer cell lines using the mirVana miRNA isolation kit (Applied Biosystems, Foster City, CA, USA). MiRNA microarray analyses were conducted by Toray Industries (www.toray.com: Tokyo, Japan). This microarray chip contains probe regions that detect 1719 miRNA transcripts listed in Sanger miRBase Release 17.0 (http://www.sanger.ac.uk). The average values of the various miRNA signal intensities detected by the chip were then compared.

### Quantitative RT–PCR

Expression levels of genes were analyzed by TaqMan quantitative RT–PCR assay for *miR-1246*, *miR-302a*, *Oct3/4* and *Sox2* (Applied Biosystems) in accordance with the manufacturer's instructions. Quantitative analysis was performed using the CFX96 Real-Time System (BioRad, Hercules, CA, USA). *U6* was used as an internal control for *miR-1246* and *miR-302a*. *GAPDH* was used as an internal control for *Oct3/4* and *Sox2*. For control of *Oct3/4* and *Sox2* expression after spheroid formation, we used AGS or HepG2 cells cultured in regular media supplemented with 10% FBS. Experiments were carried out in triplicate.

### Antibodies

Antibodies against Girdin (T-13) and CDK2 (SC-163) were purchased from Santa Cruz Biotechnology (Dallas, TX, USA). Antibodies against DYRK1A (Cell Signaling, Danvers, MA, USA), EZH2 (BD Biosciences, San Jose, CA, USA) and BMI-1 (Abcam, Cambridge, UK) were used. HRP-conjugated β-actin (Santa Cruz Biotechnology) was used as an internal control for western blotting. ChIP Ab+ EZH2 (Millipore, Billerica, MA, USA) and p53 antibody (Cell Signaling) were used for the ChIP assay.

### Chromatin immunoprecipitation (ChIP) assay

The ChIP assay was performed as described previously.^12^ Quantitative analysis was performed using the CFX96 Real-Time System (BioRad) with Ssoadvanced SYBR Green SuperMix (BioRad). The sequences of the primers used were as follows:

*miR-1246* forward: 5′-GCTTGGCTAGCTGCCTTAACA-3′

*miR-1246* reverse: 5′-GGATCTGCAGGCACTAACTTGTAG-3′.

*miR-302a* forward: 5′-GGGTAAAAGGCAGGGACTTC-3′,

*miR-302a* reverse: 5′-CAGACCCACCCAGGATCATA-3′.

*miR-4448* forward:5′-TATTACCTGCGTCCGAGGAG-3′

*miR-4448* reverse:5′-CTCGTGCTCTATGGTGTTGC-3′

The fraction of immunoprecipitated DNA was calculated as follows: (DNA immunoprecipitated with EZH2 or p53 antibody—negative control with IgG)/(input DNA—negative control with IgG). Experiments were carried out in triplicate.

### Western blotting

Protein extracts were separated by SDS/polyacrylamide gel electrophoresis and transferred to nitrocellulose membranes, and the membranes were hybridized with the antibodies described above. β-Actin was used as an internal control.

### Apoptosis assay

AGS and HepG2 cells were cultured in media supplemented with 10% FBS and treated with 1 μM SAHA and 5 μM DZNep for 72 h. Apoptosis assay was performed using an Annexin V-FITC Apoptosis Detection Kit (Bio Vision, Milpitas, CA, USA). Flow cytometry analysis was performed using BD FACSDiva (BD Bioscience). The data are indicated as mean±s.d. for three independent experiments.

### Cell cycle assay

AGS and HepG2 cells were cultured in media supplemented with 10% FBS and treated with 1 μM SAHA and 5 μM DZNep for 72 h. Cells were harvested by trypsinization, washed with PBS, and then fixed in 70% ice-cold ethanol overnight at 4 °C. They were then washed with PBS and treated with RNase A (1 mg/ml) at 37 °C for 60 min and incubated with propidium iodide (50 μg/ml) for 30 min at room temperature. After incubation, flow cytometry analysis was performed using a BD FACSDiva (BD Bioscience).

### Sphere-forming assay

For sphere-forming assay, we used ReproFF2 medium (Repro cell, Kanagawa, Japan), which is a new feeder-free culture medium for human ES/iPS cells. We also used the Ultra-low attachment surface (Corning, Corning, NY, USA), which is coated with a hydrogel layer and prevents attachment of cancer cells growing in an anchorage-dependent manner.^40^ AGS and HepG2 cells were cultured in serum-free ReproFF2 medium containing 5 ng/ml bFGF with 5 μM DZNep or 1 μM SAHA in a 96-well plate with an ultra-low attachment surface. The AGS and HepG2 cells were plated at 200 and 500 per well, respectively. Fresh culture medium was added on day 5 of the culture period. The number of spheroids was counted 10 days after drug treatment. Experiments were carried out in triplicate.

### Wound-healing assay

The migration of AGS and HepG2 cells was measured by a wound-healing assay. AGS and HepG2 cells were seeded and grown to ∼80% confluence in medium supplemented with 10% FBS and treated with 1 μM SAHA and 5 μM DZNep for 72 h. Then a wound was created across the center of the well by scratching with the tip of a 1000-μl pipette. The wells were then washed twice with medium and replenished with fresh culture medium containing 5 μM DZNep. The cells were then allowed to migrate for 72 h, after which they were fixed with 3.7% paraformaldehyde and stained with 1% Crystal Violet in 2% ethanol. Three randomly selected sites per well were photographed, and the area of the wound was quantified using Image J software. The migration of cells was evaluated from the width of the wounded area or by counting the number of migrated cells. Experiments were performed in triplicate.

### Statistical analysis

For statistical analysis, the unpaired *t*-test was used. Differences at *P*<0.05 were considered significant. All error bars represent s.d.

## Figures and Tables

**Table 1:** MiRNA expression profiles in AGS and HepG2 cells treated with SAHA and DZNep

	*AGS-SAHA*				*HepG2-SAHA*		
	*Cont*	*SAHA*	*Ratio*		*Cont*	*SAHA*	*Ratio*
*miR-302a	28.4	410.6	14.4	*miR-301a*	32.3	258.7	8.0
*miR-205*	19.7	210.0	10.7	*miR-16*	177.5	1187.2	6.7
*miR-1915*	34.3	300.2	8.8	*miR-4286*	3189.0	20115.4	6.3
*miR-146b-5p*	38.1	265.3	7.0	*miR-378c*	28.5	158.0	5.5
*miR-188-5p*	21.5	143.8	6.7	*miR-107*	649.3	3513.9	5.4
*miR-221*	139.6	896.9	6.4	*miR-103*	895.0	4614.8	5.2
*miR-663*	84.5	532.5	6.3	*miR-18a*	91.7	470.1	5.1
*miR-2861*	325.6	1860.0	5.7	*miR-125a-3p*	107.7	540.7	5.0
*miR-181a*	57.0	320.7	5.6	*miR-15a*	31.5	155.0	4.9
*miR-3196*	437.5	2322.5	5.3	*miR-378*	61.1	281.4	4.6
*miR-1469*	73.2	383.6	5.2	*miR-422a*	52.7	187.4	3.6
*miR-3621*	210.6	1079.1	5.1	miR-21*	39.2	135.9	3.5
*miR-3180*	45.3	229.9	5.1	*miR-19b*	349.3	1126.3	3.2
*miR-3656*	295.0	1469.4	5.0	*miR-22*	501.9	1609.3	3.2
*miR-1908*	221.2	1083.9	4.9	*miR-532-5p*	34.9	107.4	3.1
*miR-4294*	63.6	310.7	4.9	*miR-660*	53.3	159.7	3.0
*miR-1268*	124.4	590.8	4.7	*miR-1246	2438.0	7229.7	3.0
*miR-3195*	34.9	164.1	4.7	*miR-185*	95.6	268.3	2.8
*miR-193b*	37.0	173.2	4.7	*miR-425*	216.4	591.1	2.7
*miR-3665*	575.6	2686.4	4.7	*miR-1260b*	17758.0	46685.1	2.6
*miR-23a*	801.3	3358.3	4.2	*miR-4284*	8795.4	22991.7	2.6
*miR-1246	820.5	3435.9	4.2	*miR-128*	74.2	193.0	2.6
*miR-373*	5716.4	23818.2	4.2	*miR-194*	1072.3	2787.3	2.6
*miR-4327*	36.3	149.3	4.1				
*miR-638*	242.2	989.9	4.1				
*miR-762*	547.8	2226.8	4.1				

	*AGS-DZNep*				*HepG2-DZNep*		
	*Cont*	*DZNep*	*Ratio*		*Cont*	*DZNep*	*Ratio*
*miR-4448	64.2	2725.7	42.4	*miR-4448	72.5	592.9	8.2
*miR-3676*	41.1	160.7	3.9	*miR-4485*	226.5	956.1	4.2
*miR-221*	141.9	393.5	2.8	*miR-4294*	95.0	339.2	3.6
*miR-1246	834.2	2299.4	2.8	*miR-3676*	120.4	416.2	3.5
*miR-3131*	35.4	93.4	2.6	*miR-4419b*	13.8	46.8	3.4
*miR-3194-5p*	14.4	34.9	2.4	*miR-4725-3p*	103.0	339.7	3.3
*miR-940*	20.4	47.8	2.3	*miR-1246	5544.7	17245.6	3.1
*miR-4730*	45.7	99.6	2.2	*miR-1290*	144.0	443.1	3.1
*miR-4745-5p*	71.9	148.2	2.1	*miR-4675*	20.0	58.3	2.9
				*miR-146b-5p*	61.6	179.1	2.9

Signal intensities and their ratios in microarray analyses are shown. MiR-1246, miR-302a and miR-4448 are indicated by asterisks.
